# Cynomolgus Macaque as an Animal Model for Severe Acute Respiratory Syndrome

**DOI:** 10.1371/journal.pmed.0030149

**Published:** 2006-04-18

**Authors:** James V Lawler, Timothy P Endy, Lisa E Hensley, Aura Garrison, Elizabeth A Fritz, May Lesar, Ralph S Baric, David A Kulesh, David A Norwood, Leonard P Wasieloski, Melanie P Ulrich, Tom R Slezak, Elizabeth Vitalis, John W Huggins, Peter B Jahrling, Jason Paragas

**Affiliations:** 1Infectious Diseases Department, National Naval Medical Center (NNMC), Bethesda, Maryland, United States of America; **2**Virology Division, United States Army Medical Research Institute of Infectious Diseases (USAMRIID), Fort Detrick, Maryland, United States of America; **3**Radiology Division, National Naval Medical Center (NNMC); Bethesda, Maryland, United States of America; **4**Department of Epidemiology, University of North Carolina at Chapel Hill, Chapel Hill, North Carolina, United States of America; **5**Diagnostic Systems Division, United States Army Medical Research Institute of Infectious Diseases (USAMRIID), Fort Detrick, Maryland, United States of America; **6**Lawrence Livermore National Laboratory, Livermore, California, United States of America; **7**Headquarters Division, United States Army Medical Research Institute of Infectious Diseases (USAMRIID), Fort Detrick, Maryland, United States of America; The University of Hong KongChina

## Abstract

**Background:**

The emergence of severe acute respiratory syndrome (SARS) in 2002 and 2003 affected global health and caused major economic disruption. Adequate animal models are required to study the underlying pathogenesis of SARS-associated coronavirus (SARS-CoV) infection and to develop effective vaccines and therapeutics. We report the first findings of measurable clinical disease in nonhuman primates (NHPs) infected with SARS-CoV.

**Methods and Findings:**

In order to characterize clinically relevant parameters of SARS-CoV infection in NHPs, we infected cynomolgus macaques with SARS-CoV in three groups: Group I was infected in the nares and bronchus, group II in the nares and conjunctiva, and group III intravenously. Nonhuman primates in groups I and II developed mild to moderate symptomatic illness. All NHPs demonstrated evidence of viral replication and developed neutralizing antibodies. Chest radiographs from several animals in groups I and II revealed unifocal or multifocal pneumonia that peaked between days 8 and 10 postinfection. Clinical laboratory tests were not significantly changed. Overall, inoculation by a mucosal route produced more prominent disease than did intravenous inoculation. Half of the group I animals were infected with a recombinant infectious clone SARS-CoV derived from the SARS-CoV Urbani strain. This infectious clone produced disease indistinguishable from wild-type Urbani strain.

**Conclusions:**

SARS-CoV infection of cynomolgus macaques did not reproduce the severe illness seen in the majority of adult human cases of SARS; however, our results suggest similarities to the milder syndrome of SARS-CoV infection characteristically seen in young children.

## Abstract 2

Jason Paragas and colleagues report the first findings of measurable clinical disease in nonhuman primates infected with the virus that causes SARS, SARS-CoV.

## Introduction

In late 2002, Chinese health officials reported an unusual number of atypical pneumonia cases in Guangdong Province, and within 2 months, the World Health Organization (WHO) was alerted of an outbreak widespread throughout the province [ 1]. By March 2003, the illness designated severe acute respiratory syndrome (SARS) had spread to Hong Kong, Singapore, Vietnam, and Toronto, Canada [ 2]. The global SARS outbreak ended that July, but during its existence the disease caused 774 fatalities and had a significant economic impact on Southeast Asia [ 3– 5]. A special concern was its predilection for nosocomial spread, as 21% of SARS cases occurred in healthcare workers . In certain local outbreaks, hospital staff accounted for over 50% of cases, and nosocomial spread to other patients or family members accounted for a significant proportion of SARS cases [ 6].

Developing adequate animal models for SARS is a high research priority. Coronaviruses tend to have a limited range of host species, but several animal species have been found to support SARS-associated coronavirus (SARS-CoV) replication [ 7]. BALB/c mice were shown to support high levels of viral replication in the respiratory tract after intranasal challenge, and this model has been used to test SARS vaccines [ 8– 10]. Infection of an additional rodent model (the strain 129SvEv mouse) showed infection of the respiratory epithelium after intranasal SARS-CoV challenge [ 11]. Domestic cats and ferrets support viral replication after intratracheal challenge, and ferret models have been used to study active and passive immunization against SARS [ 12– 14]. Research has focused on several animal species as potential natural reservoirs for SARS-CoV. Himalayan palm civets *(Paguma larvata)* and raccoon dogs *(Nyctereutes procyonoides)* were found to be susceptible to infection with a virus closely related to human SARS-CoV [ 15]. Experimental infection of civets produced clinical illness and histopathological evidence of pneumonia [ 16]. Chickens and pigs challenged with SARS-CoV had viral RNA in blood during the first week postinfection, but neither species appeared to support significant viral replication or manifested clinical illness [ 17]. Recently, Li et al. [ 18] reported that several species of wild bats in China are carriers of a coronavirus closely related to SARS-CoV. No studies have evaluated animal model infection or pathogenesis of recombinant infectious clone SARS-CoV (icSARS-CoV) derived from a molecular clone [ 19].

Nonhuman primate (NHP) models of SARS-CoV infection have yielded absent to moderate observable disease that has not replicated the severity of human SARS [ 20– 25]. Fever was notably absent in all studies, except for one African green monkey on day 3 postinfection [ 20]. All studies detected SARS-CoV replication in one or several monkey species and documented seroconversion, thereby confirming established infection. Aside from observable clinical symptoms, these studies relied on virus shedding and histopathology specimens from necropsy as objective markers of disease. Most studies euthanized animals during the course of infection to document histopathological disease. Only two studies followed animals for more than 14 d after infection [ 20, 22]. No study has examined radiographic evidence of pulmonary disease, which is one of the most prominent features of SARS in humans.

In adult humans, SARS presents as a severe febrile pneumonia [ 1]. It has been characterized as a three-phase illness: a first phase consisting of a flu-like illness, followed by a phase of lower respiratory tract disease, with a third phase of clinical deterioration in a process resembling adult respiratory distress syndrome [ 26]. Disease progression can be somewhat slow, with onset of severe respiratory disease occurring anywhere from 1 to 2 wk after initial symptoms [ 27]. Pulmonary radiographic abnormalities are almost universally reported in SARS cases [ 28]. However, early radiographs may be normal, and there is clear evidence of infection without radiographic abnormality in a small number of cases [ 29, 30]. Multifocal disease is present in 30–50% of initial radiographs, and the majority of persons progress to multifocal disease that peaks between 8 and 14 d after symptom onset [ 28, 31– 34]. Severe disease develops in up to 30% of patients, with the most ill developing diffuse or confluent airspace consolidation consistent with adult respiratory distress syndrome [ 28, 31, 33].

In contrast to adults, SARS in young children tends to be a relatively mild disease [ 35]. Adolescents can experience significant respiratory disease similar to adults, but younger children generally do not [ 36– 39]. Constitutional symptoms such as myalgias, chills, and headache that are common in adults are usually absent in children [ 35, 40]. Children have a shorter course of illness, most being afebrile by 7 d, and generally do not develop pulmonary disease significant enough to require assisted ventilation or even supplemental oxygen [ 36– 39, 41]. As a result, the WHO diagnostic criteria were not reliable in identifying SARS in pediatric patients [ 38]. Some experts have recommended the term “mild acute respiratory syndrome” for SARS-CoV infection in children [ 35].

Radiographic findings in children with SARS are also less significant than in adults, in both presentation and progression [ 40]. Up to 50% of children have normal initial chest radiographs [ 35]. In children with abnormal radiographs, unilateral, focal airspace disease predominates [ 36, 37, 39]. Most children have worsening of radiographic disease as illness progresses, with multifocal or bilateral lung involvement developing in 20–50% of cases [ 39, 42]. Radiographic abnormalities in children generally resolve quickly, within 6–14 d [ 37, 42, 43].

In this report, we document the results of observational studies of SARS-CoV and icSARS-CoV infection in nonhuman primates. These studies focused on clinical and virologic parameters associated with infection in cynomolgus macaques in an attempt to examine the underlying mechanism of disease and to study a potential animal model for SARS.

## Methods

### Virus and Cells

SARS-CoV Urbani strain was obtained from the Centers for Disease Control and Prevention (Tom Ksiazek) in Atlanta, Georgia and had been passaged four times in Vero E6 cells (American Type Culture Collection, Manassas, Virginia, United States) before inoculation. Final preparations of SARS-CoV in PBS that were used in infection procedures (below) had a titer of 3 × 10 ^6^ plaque-forming units (pfu)/ml. SARS-CoV-specific antibody titers were determined by plaque reduction neutralization test (PRNT) on the day of infection and at 8 wk postchallenge. PRNTs were performed on confluent Vero cells in 6-well plates as described [ 9]. Recombinant virus (icSARS-CoV) was used at passage 1 past the initial transfection, and the derivation of this molecular clone has already been reported [ 19]. The icSARS-CoV preparation titered at 1.25 × 10 ^6^ pfu/ml.

### Animals and Procedures

Eight (one female and seven male) adult cynomolgus macaques *(Macaca fascicularis)* weighing between 4.6 and 8.8 kg were infected (see below) with SARS-CoV in two separate iterations ( *n* = 2 and *n* = 6) of the experiment. Animals were observed daily for 20 d postinfection. In the six animals that constituted the second iteration, additional blood was drawn on day 26 postinfection.

Animals were anesthetized for physical examination, and blood was drawn once preinfection (on day minus 3 or 2) and then every other day beginning on the day of infection (day 0). Anesthesia was achieved by intramuscular injection of Telazol at 3 mg/kg. Anesthetized animals received a physical examination, chest radiographs, saphenous vein blood draw, and swabs of oral, nasal, and rectal mucosa. Feces were collected from animal cages on blood draw days. A metabolic pan with a stop-valve drain was used to collect urine.

Experiments were conducted in biosafety level-3 laboratories. For procedures in animal rooms, personnel wore Tyvek suits with double gloves and a powered air-purifying respirator. Personnel handling the virus or potentially contaminated clinical specimens in the laboratory used a Class II biological safety cabinet while wearing a powered air-purifying respirator.

All research was conducted in compliance with the Animal Welfare Act and other federal statutes and regulations relating to animals and experiments involving animals and adhered to principles stated in the Guide for the Care and Use of Laboratory Animals. This research was conducted in a facility fully accredited by the Association for Assessment and Accreditation of Laboratory Animal Care International.

### Infection

NHPs were infected by various routes. Intranasal (IN) infection consisted of 0.5 ml of virus preparation inoculated directly into each nostril by syringe. Conjunctivial (CJ) infection consisted of 0.5 ml inoculated into the inferior conjunctival fornix of each eye. Intravenous (IV) infection was achieved by direct injection of 1.0 ml into the saphenous vein. For intrabronchial (IB) infection, a pediatric bronchoscope was passed into the right mainstem bronchus, and 4 ml of virus preparation was instilled.

Animals were infected in three groups. Group I ( *n* = 4) animals were inoculated in the nares and bronchus (IN/IB), group II ( *n* = 2) in the nares and conjunctiva (IN/CJ), and group III ( *n* = 2) intravenously (IV). All infections were carried out under anesthesia as described above. Two animals (292Q and 91–379) in group II were infected with the icSARS-CoV, and the rest of the animals were infected with wild-type virus.

### Radiographs

Upright anterior-posterior (AP) and lateral chest radiographs were performed while animals were under anesthesia. Digital radiographs were taken with a MinXray HF 100/300 (MinXray, Northbrook, Illinois, United States) portable X-ray machine. Radiographs were digitally processed with a Fuji FCR XG-1 Smart CR reader using Fuji Synapse software (FUJIFILM Medical Systems USA, Stamford, Connecticut, United States). Radiographs were read by a board-certified chest radiologist (ML) who was blinded to route of infection and clinical course of animals.

### Hematology and Clinical Chemistries

Total WBC count, lymphocyte count (absolute and percent), hemoglobin, hematocrit, platelet count, and red cell indices were measured with a COULTER Ac·T hematology analyzer (Beckman Coulter, Fullerton, California). Albumin, alkaline phosphatase, ALT, AST, amylase, BUN, creatinine, total bilirubin, total protein, calcium, cholesterol, and glucose were measured with a Vetscan blood chemistry analyzer (Abaxis, Union City, California, United States).

### Virus Genome Detection

Two real-time reverse transcription polymerase chain reaction (Q-PCR) assays were developed to detect viral RNA in NHP biosamples ( Tables S1 and S2). These assays targeted SARS-CoV genes *POL* and *SMP* respectively. Details of development and testing of the SARS-CoV-specific Q-PCR assays ( Table S1) can be found in Protocol S1. RNA was extracted from NHP biosamples using Trizol (Invitrogen, Carlsbad, California, United States) according to the manufacturer's recommendations. Purified RNA was analyzed on the Ruggedized Advanced Pathogen Identification Device (RAPID, Idaho Technology, Salt Lake City, Utah, United States) as follows: 5 μl of RNA from each sample was added to the appropriate SARS-specific assay master mix (15 μl) and cycled in the LightCycler as described in Protocol S1. All test runs included at least one positive control that contained 0.075 pfu equivalents of plaque-purified SARS-CoV RNA and two no-template controls: reagent and sample. Quantified Q-PCR results were reported for the POL TaqMan assay.

### Virus Isolation

Virus isolation was attempted from selected biosamples from the second iteration of six animals. Specimens with a Q-PCR crossing threshold (CT) of less than 32 CT underwent culture for viral isolation. Briefly, 200 μl of media from swab samples was used to inoculate T25 flasks of Vero E6 cells ( ATCC). Flasks were monitored daily for cytopathic effect. Viral culture supernatants were prepared and titered using viral plaque assay. Cells were scraped, pelleted, and fixed in 2% glutaraldehyde in 0.1 M Millonig's phosphate buffer. Cell pellets were washed, postfixed in 1% osmium tetroxide, dehydrated and embedded in Poly/Bed 812 resin (Polysciences, Warrington, Pennsylvania, United States). Ultrathin sections were stained with uranyl acetate and lead citrate and examined by transmission electron microscopy for virions.

## Results

### Clinical Observations

We infected eight cynomolgus macaques with SARS-CoV in three groups: Group I ( *n* = 4) was infected in the nares and bronchus, group II ( *n* = 2) in the nares and conjunctiva and group III ( *n* = 2) intravenously. Basic clinical findings are summarized in Table 1. All animals in groups I and II displayed mild to moderate symptoms of illness beginning between postinfection days (PID) 2–4. Observed symptoms included decreased activity, decreased feeding, snuffling, and mildly labored breathing. Animal activity and appearance returned to baseline by PID 12–14 in all cases. Animals infected with wild-type SARS-CoV or with icSARS-CoV both displayed clinical signs of illness. We observed no overt clinical illness in the group III animals, which were infected intravenously.

**Table 1:**
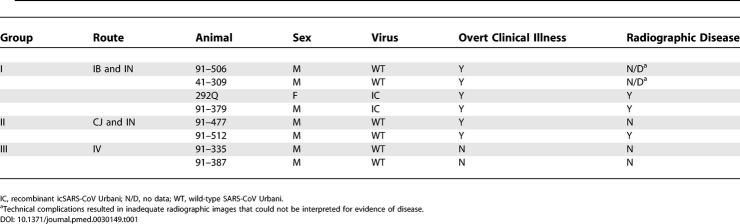
Summary of Routes of Infection and Clinical Findings

None of the animals developed a measurable fever. Continuous telemetry monitoring in four animals revealed no significant changes in temperature, heart rate, blood pressure, or left ventricular pressures (unpublished data).

### Radiographs

**Figure 1:**
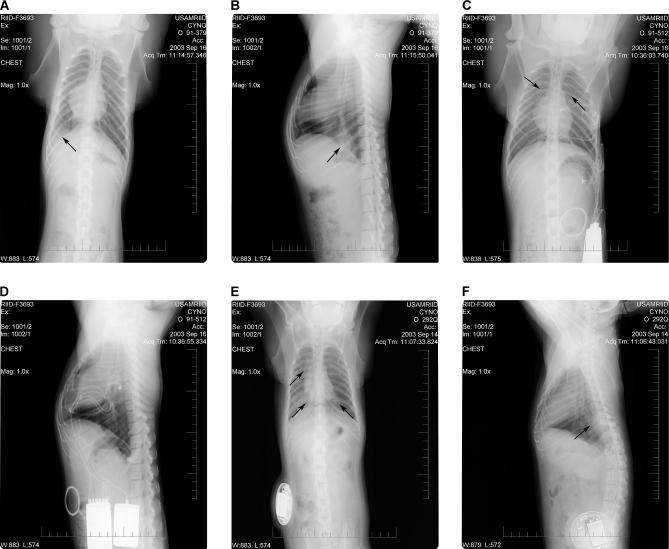
Chest Radiographs Anterior-posterior (AP) (A) and lateral (B) chest radiographs from 8 d postinfection for animal 91–379 show focal airspace disease in the right lower lobe that obscures the right costophrenic angle. Air bronchograms can be seen on the lateral view. Radiographs showing AP (C) and lateral (D) views from day 8 for animal 91–512 demonstrate subtle bilateral perihilar airspace disease visible on the AP film. Radiographs of animal 292Q on day 6 (E, F) reveal airspace disease involving the right lower lobe, left lingula, and perihilar right upper lobe. Lower lobe disease is clearly seen on the lateral view (F).

Chest radiograph results were available for six of the animals. Three of them exhibited radiographic evidence of pulmonary disease. The two animals infected by the IV route (group III) had no significant change in chest radiographs, while three of four animals infected by a mucosal route (groups I and II) developed radiographic disease ( Figure 1). Two animals with radiographic disease were infected with the icSARS-CoV (both group I) and one animal (group II) was infected with wild-type virus.

Of the three animals with radiographic evidence of disease, two (292Q and 91–379) were infected by the IB/IN route (group I) and one (91–512) was infected by the CJ/IN route (group II). All developed significant airspace disease on PID 6. The group II animal had normal radiographs until PID 6, when it developed a perihilar left upper lobe infiltrate. By PID 8, there was perihilar disease in the right upper lobe as well. These findings were relatively subtle and began to resolve on PID 12. They were still evident, albeit continuing to resolve, by PID 18. The two group I animals had subtle changes on PID 2 (right mid-lower lobe for 292Q, right lower lobe for 91–379). Radiographic appearance was unchanged on PID 4 in 292Q, but 91–379 did have a slight increase in the right lower lobe opacity. On PID 6, both animals had dramatic radiographic changes, with 292Q developing increasing lower lobe disease and new right-upper lobe airspace disease and 91–379 showing a significant increase in the right lower lobe consolidation. Both 292Q and 91–379 had increasing disease on PID 8 and demonstrated slight improvement by PID 10. Changes completely resolved by PID 14 in 91–379, but mild residual and resolving changes were still evident in 292Q on PID 18.

### Hematology

Blood cell counts reflected only minimal changes. Infected animals did demonstrate a statistically significant trend of rising total leukocyte (WBC) count during the course of infection ( Figure S1A– S1C). Mean WBC count rose from 5.5 × 10 ^9^ per liter on day 0 to a peak of 10.6 × 10 ^9^ per liter on PID 12 ( *p* = 0.006, two-tailed paired t-test), but no animal had a WBC count above the USAMRIID upper limit of normal of 15.0 × 10 ^9^ per liter. This trend in WBC count was observed across all groups, regardless of route of exposure. Absolute and percent lymphocytes did not follow a consistent trend ( Figure S1D– S1I). Several aberrant platelet counts were noted, thought to be secondary to clotting or platelet clumping. Overall, no significant change in platelet count was observed for any group. No abnormal trends were noted in other hematologic parameters ( Figure S1J– S1O).

### Serum Chemistries

Alkaline phosphatase ( Figure S2A– S2C) became elevated in two group I animals, peaking at 450 units per liter (USAMRIID upper limit of normal = 288 U/l) on day 12 for one (41–309) and at 340 units per liter on day 18 for the other (292Q). Alkaline phosphatase was elevated at two consecutive data points for both animals and had returned to normal by day 26. Other liver-associated parameters did not reflect any hepatic injury or dysfunction in these animals ( Figure S2D– S2I). One animal (91–506) in group I had an isolated elevated alanine aminotransferase (ALT) of 229 units per liter (USAMRIID upper limit of normal = 85 U/l) on day 2 ( Figure S2D). The ALT was within normal range on every other day for this animal, and other liver-associated parameters remained within normal limits during the course of infection. Serum blood urea nitrogen (BUN) and creatinine did not suggest a significant change in renal function in any animal during the course of SARS-CoV infection ( Figure S2J– S2O).

### Neutralizing Antibodies

All animals had convalescent anti-SARS-CoV antibodies detected by plaque reduction neutralization test (PRNT performed 8 wk postinfection. Neutralizing antibodies were undetectable at baseline in all animals. PRNT _50_ results are shown in Table 2. Group I animals had higher titers of neutralizing antibody, but comparison of geometric mean titers between groups by analysis of variance showed no statistical difference.

**Table 2:**
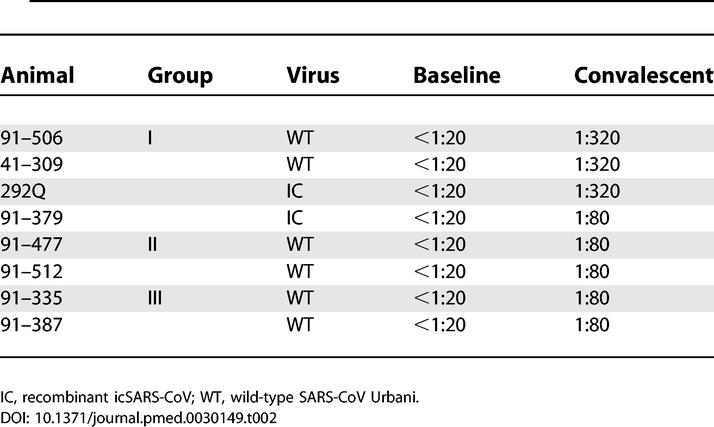
PRNT _50_ Antibody Titers at Baseline (Day 0) and During Convalescence (8 wk Postinfection)

### Viral Detection in Biosamples

Viral RNA was detected by real-time Q-PCR in biosamples taken from all animals ( Table 3). In particular, viral genome in nasal swabs and urine was detected in all animals regardless of route of exposure. Viremia was not a common finding. The two group III (infected IV) animals had virus detected in blood shortly after inoculation, as would be expected, but only one of these animals (91–335) had detectable viremia after day 0. From either group I or II only one animal (91–512) had detectable viremia, with positive Q-PCRs on PID 8 and 20.

**Table 3:**
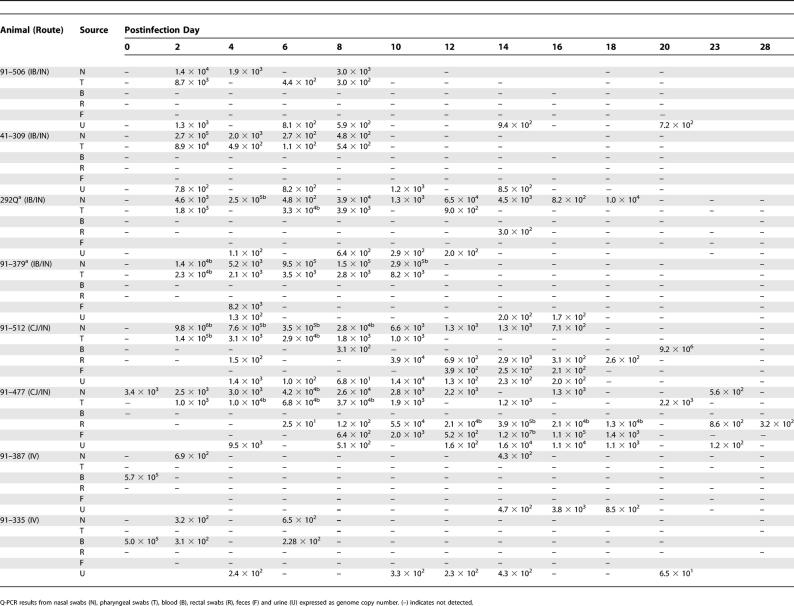
Viral Genome Detection and Isolation

Virus shedding in the urine was universal, detected as early as PID 2 and as late as PID 23. Urinary excretion of virus was a predominantly late phenomenon. Shedding from the nasopharynx was detected in all animals, although it was more common in animals challenged by mucosal route versus intravenous route (average number of days, 6.8 versus 2, *p* = 0.041, ANOVA). One animal (91–477) had a positive nasal swab on day 0. This result was probably due to cross-contamination at the time of collection. Three animals (292Q, 91–477, and 91–512) that had the most prolonged and persistent nasal shedding had been inoculated by mucosal routes.

Detection of viral RNA in rectal or fecal specimens varied with the route of infection ( Table 3). Group II animals had viral genome detected in at least five contiguous rectal swabs or fecal specimens (spanning 10 d) and had positive specimens on an average of 7.5 d. Viral RNA was found in two out of four of the group I animals (1 d each) and was undetected in group III animals ( *p* = 0.001, ANOVA).

A total of 30 biosamples with a Q-PCR crossing threshold under 32 from five animals were cultured. Live virus was isolated from 20 of these samples ( Table 3). Two samples with crossing threshold over 32 and one negative control specimen had negative culture results. Both wild-type and infectious clone SARS-CoV were isolated from culture. Transmission electron micrography of positive cell cultures revealed spherical virions on the surface of cells as well as maturing virions budding into smooth-walled vesicles consistent with a coronavirus ( Figure S3).

## Discussion

This study demonstrated that cynomolgus macaques infected with SARS-CoV develop clinical disease with pulmonary radiographic findings. The presence of radiographic disease appeared to depend upon the route of infection. Of four animals challenged by a mucosal route (groups I and II), three had radiographic evidence of pulmonary disease. Radiographic disease developed by PID 6, and it peaked and resolved rapidly. Neither animal in the intravenous challenge group (group III) developed radiographic disease.

SARS-CoV replication was detected at a variety of sites in infected animals. The virus displayed tropism for the upper respiratory mucosa, but replication at distal sites reflects systemic viral infection. Data from human studies suggest that nasopharyngeal shedding of SARS-CoV increases for the first 10 d of symptoms [ 26]. We detected no consistent pattern in nasopharyngeal shedding. Viremia was not a prominent feature and was found in only one of the intravenously infected animals at days 2 and 6.

In our study, frequency and source of SARS-CoV genome detection correlated with route of inoculation. Animals infected intravenously (group III) had a paucity of positive specimens. Viruria appearing after day 10 was the only persistent finding in these animals ( Table 3). Animals infected mucosally (groups I and II) had a greater number of positive specimens from nasal swabs and throat swabs. The two group II animals had the most positive specimens and were the only animals to have significant fecal or rectal shedding. Presence and degree of viral shedding did not appear to correlate with radiographic disease. The group II animal (91–477) that did not develop radiographic disease had extensive viral shedding from the nasopharynx, urine, and gastrointestinal tract.

No unusual changes in hematologic and serum chemistry parameters were observed ( Figures S1 and S2). WBC count increased significantly but remained within our laboratory's normal range. Unlike experience in humans and other studies with NHPs, we did not detect a measurable change in lymphocyte or platelet counts [ 22, 23, 25, 44– 47]. Renal and hepatic markers did not suggest significant derangement during the course of infection, although alkaline phosphatase was elevated briefly in two animals ( Figure S2A). We cannot explain the origin of this transient elevation in alkaline phosphatase, but the absence of abnormalities in other liver-associated enzymes and the brief duration of the abnormal values make significant hepatic or bone injury unlikely.

Disease produced by wild-type SARS-CoV Urbani and by icSARS-CoV was clinically indistinguishable. Observable symptoms and laboratory parameters did not vary between the two viruses. Both icSARS-CoV-challenged animals developed radiographic disease. Of the two animals infected with wild-type virus by a mucosal route, only one developed radiographic disease, and radiographic findings were relatively subtle. We cannot exclude the possibility that icSARS-CoV produces more significant pulmonary disease, but the difference in findings is more likely related to route of infection and dose. Animals that received icSARS-CoV (both in group I) were infected in the bronchus with a higher dose of virus. Importantly, the fact that recombinant icSARS-CoV produced disease clinically similar to its Urbani parent supports the hypothesis that the molecular clone recapitulates the disease phenotype in the macaque. These findings suggest that SARS-CoV is the sole cause of SARS, without the involvement of coinfecting or passenger agents. Models using icSARS-CoV may pave the way for identification of genetic determinants of pathogenesis.

All animals seroconverted in convalescence, with neutralizing antibodies detected 8 wk after infection ( Table 2). Groups I and II had higher neutralizing antibody titers than group III. This difference was not statistically significant, perhaps due to the low number of animals involved. Infection did appear to impart protective immunity, at least in the short term. Six animals were rechallenged by IN and CJ inoculation of SARS-CoV 13 wk after initial infection. All six displayed no clinical signs of disease and had normal chest radiographs (unpublished data). These six animals included the two animals initially challenged with the recombinant icSARS-CoV, demonstrating that molecularly cloned virus induced protective immune responses against its wild-type parent.

Our study has some potential limitations. This was an observational study that was not designed to achieve statistical significance in all parameters measured. We infected a small number of animals by each route, so we may not have observed the entire spectrum of disease severity [ 29, 30, 48]. In order to ensure virus delivery to lower respiratory mucosa, we instilled a larger volume of virus preparation through the bronchoscope to group I animals. Thus group I received a total inoculum 2.5 times that of group II and five times that of group III. Finally, we used a relatively high inoculum of virus. Although the infectious dose (ID _50_) in humans is unknown, it is likely that the dose we administered is significantly larger. We used this high inoculum with the intention of producing a more uniform presentation of disease in the study animals.

Our experience offers contrast to previously published studies of SARS-CoV infection in nonhuman primates [ 20– 23, 25]. We found mild to moderate symptomatic disease in cynomolgus macaques infected mucosally. Fouchier et al. [ 21] and Kuiken et al. [ 23] described more significant clinical disease in cynomolgus macaques, with lethargy, rash, and respiratory distress evident in some animals. Bukreyev et al. [ 22] and McAuliffe et al. [ 20] recount no overt disease in rhesus, cynomolgus, or African green monkeys challenged with SARS-CoV Urbani, but they report a higher degree of viral replication in African greens. One African green monkey was also noted to have a single febrile temperature on PID day 3 [ 20]. Rowe et al. [ 25] examined SARS-CoV infection in cynomolgus and rhesus macaques and reported only minimal symptomatic disease. In the Rowe et al. study, as in ours, symptomatic infection was detected only in animals challenged mucosally and not in animals infected IV [ 25].

Evidence of persistent viral replication in the upper airways has varied between studies. Rowe et al. [ 25] reported a sparse number of positive nasal and oral swabs, although one of each was positive at PID 12. In contrast, combined data from Kuiken et al. [ 23] and Fouchier et al. [ 21] show nasal or pharyngeal shedding in five of six animals. McAuliffe et al. [ 20] detected respiratory tract shedding for 2–10 d after infection. We found that mucosally infected animals had universal shedding from the nasopharynx between PID 2 and 8 and had frequent shedding out to PID 23. We found significant and persisting virus presence in the urine and feces as well. Other studies have not reported testing urine for virus presence, although Kuiken et al. [ 23] report virus detection by PCR at necropsy in the kidney or urinary bladder of one animal each. McAuliffe et al. [ 20] found positive fecal samples by RT-PCR only in African green monkeys. Kuiken et al. [ 23] and Rowe et al. [ 25] tested rectal swabs for presence of virus but found none positive.

Observed differences in model performance are difficult to explain but may be attributable to viral strain, dose or route of infection. Bukreyev et al. [ 22] and McAuliffe et al. [ 20] also used the Urbani strain of SARS CoV given by a similar route (intranasal and intratracheal), but our clinical observations were significantly different. We used a different assay method, but our total dose per animal was probably higher, as they used 10 ^6^ to 10 ^6.3^ cell culture infective doses (also referred to as TCID _50_). Fouchier et al. [ 21] and Kuiken et al. [ 23] used a different strain of virus isolated from patient 5688. They observed the most prominent clinical symptoms despite using a similar dose (1 × 10 ^6^ cell culture infective doses) administered in the nares and trachea. Rowe et al. [ 25] used a dose similar to ours (10 ^7^ pfu) of SARS-CoV isolated from a Canadian patient. Their route of delivery was also through the nares and trachea.

The radiographs in our study present a much clearer picture of pulmonary disease than have previous studies using NHPs. In Kuiken et al. [ 23] and Fouchier et al. [ 21], pneumonia was evident in pathological specimens from necropsy at PID 6, but animals were not followed past this point. Only two of eight animals in the study by Rowe et al. [ 25] had pathological evidence of lung disease, but necropsies were performed 14 d postinfection. McAuliffe et al. [ 20] found histopathologic evidence of viral lung infection on PID 2 that was resolving by PID 4. None of these studies used any other objective method to measure pulmonary disease.

The radiographic appearance of pneumonia in our study may afford insight into NHP models of SARS. Radiography provides an objective but dynamic measure of pulmonary disease. Our radiographic findings are similar to radiographs of humans with less-severe cases of SARS [ 28, 31, 32]. There is growing evidence that SARS may present as mild or indolent disease in adults [ 29, 30, 48]. Such mild disease in adults does appear to be an exception, however. Overall, our experience confirms that clinical disease from SARS-CoV infection in NHPs does not closely reproduce SARS in adult humans.

The similarity between our findings and SARS in children deserves further examination. We observed unifocal or multifocal radiographic disease resolving quickly instead of progressing to severe diffuse disease, which is consistent with findings in children with SARS [ 37, 39, 42, 43]. Absence of fever may not be as significant as it would seem. In published reports, children with SARS did present with fever, but the data are biased, because fever was universally included in case definitions [ 36– 39, 41]. Fever in children less than ten years old who are diagnosed with SARS tends to be relatively mild and short in duration [ 36– 39, 41]. In these reports, it is possible that children with mild clinical illness from SARS who did not develop a significant fever may have remained undiagnosed. This premise is supported by at least one documented case of SARS in an adult without fever [ 49]. Hematologic findings, particularly lymphopenia, are more prevalent in children with SARS [ 36– 39, 41] than in the macaques of our study, but Rowe et al. [ 25] did find lymphopenia and thrombocytopenia in their animals.

Understanding the differences between SARS in adults and in children may be very important in unraveling the underlying pathogenesis of SARS. Adults and children appear to have similar levels of SARS-CoV viremia associated with infection, implying that levels of viral replication are similar in both groups [ 50]. Adults with SARS have significantly increased serum levels of inflammatory cytokines and chemokines [ 51– 53]. Activation of inflammatory cytokines in children with SARS may be less dramatic [ 54], and preliminary data suggest that this is the case in NHPs as well [ 25]. Thus, immune response may account for variations in SARS severity between age groups and even species.

We have described our experiments with SARS-CoV infection by a variety of routes in cynomolgus macaques. Wild-type SARS-CoV and recombinant icSARS-CoV produced similar disease in our macaque model. As in prior studies, we found overt clinical disease in animals infected by mucosal routes that was much less severe than adult human SARS. We found radiographic pulmonary disease in most of these animals that resembled radiographic findings in human SARS. Overall, the disease we observed had some similarities to SARS in young children. Further study is needed to determine the applicability of NHP models to the study of SARS.

## Supporting Information

Figure S1 Line Graphs of Complete Blood Count Results Hematology results from cynomolgus macaques infected with SARS-CoV on day 0. Included are results for WBC count (A–C); absolute lymphocyte count (D–F); percent lymphocytes (G–I); hematocrit (J–L); and platelet count (M–O). Results are grouped by route of infection: group I, intrabronchial + intranasal (A, D, G, J, M); group II, conjunctival + intranasal (B, E, H, K, N); group III, intravenous (C, F, I, L, O).
(4.7 MB DOC)

Figure S2 Line Graphs of Serum Chemistry Results Serum chemistry results from cynomolgus macaques infected with SARS-CoV on day 0. Included are results for alkaline phosphatase (alk phos) (A–C); alanine aminotransferase (ALT) (D–F); albumin (G–I); blood urea nitrogen (BUN) (J–L), and creatinine (M–O). Results are grouped by route of infection: group I, intrabronchial + intranasal (A, D, G, J, M); group II, conjunctival + intranasal (B, E, H, K, N); group III, intravenous (C, F, I, L, O).
(5.4 MB DOC)

Figure S3 Electron Micrograph of SARS-CoV from Cell Culture Transmission electron micrograph of Vero cell infected with SARS-CoV isolate recovered from sample from infected nonhuman primates shows sphere-shaped virions on the surface of the cell as well as maturing virions budding into smooth-walled vesicles.
(3.3 MB PSD)

Protocol S1 Development of SARS-CoV-Specific Q-PCR Assays (38 KB DOC)

Table S1 Primer and Probe Sequences for SARS-CoV-Specific Q-PCR Primer and probe names, sequences, and final concentrations for two SARS-CoV specific Q-PCR assays developed to detect SARS-CoV genome in infected NHPs.
(31 KB DOC)

Table S2 Testing of SARS Real-Time PCR Assays Results of *POL*-minor binding groove (MGB) and *SMP*-MGB assays for samples spiked with SARS coronavirus versus samples spiked with non-SARS coronaviruses.
(34 KB DOC)

**Patient Summary**

**Background**

The emergence of severe acute respiratory syndrome (SARS) in Asia 2003 in many ways served as reminder of how a new infectious disease had the potential to cause huge economic disruption, as well as being of substantial public health importance. Although the cause of the disease was discovered quite quickly—a new virus known as SARS-associated coronavirus (SARS-CoV) (corona being a description of the virus's spiky, crown-like appearance when seen under an electron microscope)—experts differed in their opinion on how the disease should be treated, and no vaccine has yet been developed for it.

**Why Was This Study Done?**

Previously identified human coronaviruses caused only mild respiratory infections. Hence researchers wanted to understand exactly how this new virus caused disease. Infecting animals with the virus was a crucial part of this research. Researchers had already given the virus to a number of animal species, ranging from mice to pigs to monkeys, but none had shown symptoms that were similar to those seen in humans.

**What Did the Researchers Do and Find?**

They infected a small number of macaques with the SARS-CoV, either into the nose and throat, nose and eye, or intravenously. Some of the animals were given the virus isolated from humans infected with disease, and others were given a genetically engineered virus that was identical in sequence to this virus. The virus was able to multiply in all the macaques, and all developed antibodies to the virus—evidence of an immune response. Chest radiographs from several animals infected via the nose, throat, or eye showed pneumonia that was at its worse between days 8 and 10 after infection. Overall, giving the virus intravenously rather than by these other routes produced a less severe infection. Also, there were no differences in signs or symptoms in animals given the natural virus rather than the engineered one.

**What Do These Findings Mean?**

Although necessarily the number of animals infected was very small, the researchers were able to get some interesting results. The infected animals did not get severe disease, but rather the disease was more like that seen in infected children. Nonetheless, this animal model of disease may well be useful in developing therapies and vaccines against this illness. In addition, they showed that the engineered virus produce the same symptoms as the one isolated from humans, which suggests that no other viruses are involved in causing SARS

**Where Can I Get More Information Online?**

MedlinePlus has a page of links to SARS information:

http://www.nlm.nih.gov/medlineplus/severeacuterespiratorysyndrome.html

The CDC also has a fact sheet on SARS:

http://www.cdc.gov/ncidod/sars/factsheet.htm

The WHO has a Web site with up-to-date information on SARS:

http://www.who.int/csr/sars

## Abbreviations

ALT: alanine aminotransferase
AP: anterior-posterior
CJ: conjunctival(ly)
CT: crossing threshold
IB: intrabronchial(ly)
icSARS-CoV: infectious clone SARS-CoV
IN: intranasal(ly)
IV: intravenous
NHP: nonhuman primate
pfu: plaque-forming units
PID: postinfection day(s)
PRNT: plaque reduction neutralization test
Q-PCR: reverse transcription polymerase chain reaction
SARS: severe acute respiratory syndrome
SARS-CoV: SARS-associated coronavirus
WBC: white blood cell
