# Supplementary material for: Cynomolgus Macaque as an Animal Model for Severe Acute Respiratory Syndrome
Source: PLoS Med. 2006 Apr 18;3(5):e149. doi: 10.1371/journal.pmed.0030149 (PMC1435788; doi:10.1371/journal.pmed.0030149)
Supplement: Supplementary file 1 [file pmed.0030149.sd001.doc]

Development of SARS-CoV_Specific Q-PCR Assays

*PCR target sequences, primers and fluorogenic probes****.*** A real-time, reverse-transcription polymerase chain reaction (Q-PCR) assay for SARS-CoV was developed to detect viral nucleic acid presence in experimentally infected animals. The real-time PCR assay primers and TaqMan^®^- MGB (minor groove binder) probe sequences are listed in Table S1. The SARS-CoV RNA-directed RNA polymerase gene was aligned with the human RNA polymerase gene (GenBank Accession # AF124989) using the EMBL-EBI ClustalW (1.82) Multiple Sequence Alignment Tool ([***http://www.ebi.ac.uk/clustalw/index.html***](http://www.ebi.ac.uk/clustalw/index.html)). Regions of non-homology were identified as target sequences for each potential SARS-specific assay. One RNA polymerase primer pair (out of 60 primer pairs tested) was identified and tested with two different TaqMan-MGB probes. In addition, 102 potential primer/TaqMan probe pairs were obtained from Lawrence Livermore National Laboratories (Livermore, CA) generated via a computational method[56]. Thirty two primer pairs were synthesized and tested. Of the 32 primer pairs tested, only 3 showed good amplification of a single amplicon of the proper size. TaqMan-MGB probe testing showed that only one primer/probe pair (SMP – small membrane protein) resulted in a robust assay. All primers were synthesized using standard phosphoramidite chemistry from Invitrogen (Carlsbad, CA). The TaqMan^®^-MGB probes were synthesized by Applied Biosystems (Foster City, CA) and contained 6-carboxyfluorescein (6FAM) at the 5' end. A non-fluorescent quencher (NFQ) and the minor groove binder (MGB) protein were added to the 3' end.

*5' Nuclease PCR (TaqMan^®^-MGB) assays****.*** Using the *Primer Express* 2.0 for Windows (Applied Biosystems, Foster City, CA) software to design potential SARS-specific TaqMan^®^-MGB assays (POL-MGB and SMP-MGB), we optimized each assay as previously reported[57]. Briefly, potential primer pairs were initially tested in the LightCycler by the fluorescent dye SYBR Green I (Roche Biochemicals, Indianapolis, IA) using the Invitrogen SuperScript^TM^ One-Step RT-PCR with Platinum^©^ *Taq* plus molecular grade bovine serum albumin (BSA) at a final concentration of 25 µg/ml. The optimum primer pair was selected based on specificity and efficiency of amplification. The selected primer pair was then optimized to the final concentration (0.1 μM to 1.0 μM) with the lowest C_t_ value and the highest fluorescent signal. Next, several potential TaqMan^®^-MGB probes were tested with the optimized primer pair by varying the probe and MgSO_4_ concentrations. The final assay consisted of the primer/probe pair concentrations and reaction conditions that combined for the lowest level of detection ((LOD), the gene copy number which was detected by the assay at least 58/60 times)), lowest C_t_ value, and highest fluorescent signal-to-noise ratio. The LODs of the assays were determined using a serial 10-fold dilution of genomic RNA purified from a single plaque of SARS-CoV Urbani.

All assays were carried out in 20 µL volumes for the LightCycler with each reaction made up in Invitrogen SuperScript^TM^ One-Step RT-PCR with Platinum^©^ *Taq* PCR buffer with molecular grade BSA added to a final concentration of 25 µg/ml. The final MgSO_4_, primer and probe concentrations for each assay are also listed in **Table S1**. Thermal cycling for the LightCycler was performed as follows: one cycle at 50^o^C for 15 min and one cycle at 95^o^C for 5 min, followed by 45 cycles of 95^o^C for 1 sec and 60^o^C for 20 sec. A fluorescence reading was taken at the end of each 60^o^C step. Each reaction capillary tube was read in Channel 1 (F1) at a gain setting of 8 with data being analyzed by the LightCycler Data Analysis software (version 3.5.3). Sample curves were analyzed using the “Second Derivative Maximum” with the baseline adjustment set to *Arithmetic*.

*Assay evaluation****.*** Both the POL-MGB and the SMP-MGB assays were evaluated against available coronavirus genomic sequences (**Table S2**). Five µl of each sample was added to the appropriate SARS-specific assay LightCycler master mix (15 µl) and cycled as described above. All test runs included at least one positive control that contained 0.075 pfu equivalents of plaque-purified SARS-CoV RNA and two no-template controls (NTC): reagent NTC and sample NTC. A serial ten-fold dilution of the SARS-CoV RNA extracted from one infectious virus plaque determined the theoretical limit of detection to be one virus particle (one copy of genomic RNA), assuming approximately 10^3^ virus particles per plaque. In addition, a GenBank BLAST search of each SARS-CoV-specific primer/probe sequence was performed.

*Validation of Q-PCR Assays.* The final primer/TaqMan^®^-MGB probe assay sequences and reaction conditions for each SARS-specific assay are shown in **Table S1**. Based on previously published data, the SARS assays reproducibly detected approximately one copy of SARS genomic RNA[57]. Results from the genomic RNA LOD experiments also correlated linearly with a dynamic range of seven orders of magnitude, representing approximately .00075 to 7,500 pfu (data not shown).

Both SARS-specific assays were tested against three strains of SARS and five strains of coronavirus RNAs. The results in **Table S2** indicate that both the POL-MGB and SMP-MGB assays were capable of detecting all of the SARS genomic RNA species available. Non-SARS coronavirus RNAs were not detected by either assay. There were no false positives among the non-SARS RNA samples for either of the assays on the LightCycler. In addition, the primer and probe sequences for each assay were subjected to a BLAST search (<http://www.ncbi.nlm.nih.gov/BLAST/>). Results indicate that each sequence is 100% homologous with every completely sequenced SARS strain currently available in GenBank (102 strains). Only the p26884S-MGB probe sequence showed any cross-identity [homology (*Oryza sativa* chromosome 3 BAC OSJNBa0087M10 (accession # AC109602) and BAC OJ1523_A02 (accession # AC090874) and *Homo sapiens* chromosome 17, clone RP11-455O6 (accession # AC027601)]. Also, POL-R90 primer has a 26/27 base pair match with SARS GZ-C (accession # AY4979) and a 23/27 base pair match with SARS Sin3408L (accession # AY559097). However, the primers and probe for the SMP assay are a complete match to both SARS GC-C and Sin3408L.

*Standard curve determination for the TaqMan^®^-MGB assay.* SARS Fragment D DNA (a 6,970 bp SARS fragment which contained the entire polymerase gene cloned into the pSMART LC-Kan vector [Lucigen Corp., Middletown, WI]) was PCR-amplified using the vector-specific primers: SL1 (forward) 5’- CAG TCC AGT TAC GCT GGA GTC – 3’ and SR2 (reverse) 5’ – GGT CAG GTA TGA TTT AAA TGG TCA GT – 3’. The amplicon was agrose gel purified and quantified by OD_260/280_. The DNA amplicon samples were serially diluted (1:10) from 71.4 pg (10^7^ gene copies) to 7.14 ag (1 gene copy) and tested with the SARS POL TaqMan^®^-MGB assay in triplicate in 20 µL of Master Mix in the LightCycler (LC) 2.0 (Roche Applied Science, Indianapolis, IN). A standard curve and efficiency of the real-time PCR reactions were determined by the Absolute Quantification module of the LC 4.0 software.

The SARS POL TaqMan^®^-MGB assays resulted in positives between 10 and 100 copies of the Fragment D amplicon (1/3 at 10 copies and 3/3 at 100 copies). However, the LOD for the assay was determined to be 714 ag (100 gene copies) because this was the lowest gene copy number for which all 60 dilutions tested simultaneously produced a positive call by the “Qualitative Detection” module of the LC 4.0 software. The “Absolute Quantification” module of the LC 4.0 software determined a PCR efficiency for the SARS POL assay of 1.912. For an assay to produce unambiguous and identical results, its amplification efficiency should be as close as possible to an efficiency value of 2. Our assays’ values correspond favorably with this theoretically optimum efficiency value. The equivalent gene copy numbers of the positive samples in **Table 3 (from text)** were determined using this standard curve.
