# Supplementary material for: Cynomolgus Macaque as an Animal Model for Severe Acute Respiratory Syndrome
Source: PLoS Med. 2006 Apr 18;3(5):e149. doi: 10.1371/journal.pmed.0030149 (PMC1435788; doi:10.1371/journal.pmed.0030149)
Supplement: Supplementary file 2 [file pmed.0030149.sg001.doc]

Figure S1 A

Figure S1 B

Figure S1 C

Figure S1 D

Figure S1 E

Figure S1 F

Figure S1 G

Figure S1 H

Figure S1 I

Figure S1 J

Figure S1 K

Figure S1 L

Figure S1 M

Figure S1 N

Figure S1 O
