# Supplementary material for: Cynomolgus Macaque as an Animal Model for Severe Acute Respiratory Syndrome
Source: PLoS Med. 2006 Apr 18;3(5):e149. doi: 10.1371/journal.pmed.0030149 (PMC1435788; doi:10.1371/journal.pmed.0030149)
Supplement: Supplementary file 3 [file pmed.0030149.sg002.doc]

Figure S2 A

Figure S2 B

Figure S2 C

Figure S2 D

Figure S2 E

Figure S2 F

Figure S2 G

Figure S2 H

Figure S2 I

Figure S2 J

Figure S2 K

Figure S2 L

Figure S2 M

Figure S2 N

Figure S2 O
