# Supplementary material for: Cynomolgus Macaque as an Animal Model for Severe Acute Respiratory Syndrome
Source: PLoS Med. 2006 Apr 18;3(5):e149. doi: 10.1371/journal.pmed.0030149 (PMC1435788; doi:10.1371/journal.pmed.0030149)
Supplement: Supplementary file 4 [file pmed.0030149.st001.doc]

**Table S1: Primer/probe sequences of SARS-CoV specific Q-PCR assays.**  Primer and probe names, sequences and final concentrations for two SARS-CoV specific real-time, reverse transcriptase PCR assays developed to detect SARS-CoV genome in infected non-human primates.

| **Primer/Probe** | **Sequences^1^** | **Final Conc.** | **MgSO_4_** |
| --- | --- | --- | --- |
| POL-F01 | 5’ – CAG AGC CAT GCC TAA CAT GCT - 3’ | 0.5 M | 5 mM |
| POL-R90 | 5’ – TAG AAA CGG TGT GAT AAG TTA CAG CAA - 3’ | 0.5 M |  |
| POLp46S-MGB | 5’ - 6FAM – TGC TCG CAA ACA TAA C – MGBNFQ - 3’ | 0.1 M |  |
| SMP-F26859 | 5’ – CCT AGG GCG CTG TGA CAT TA - 3’ | 0.5 M | 5 mM |
| SMP-R26974 | 5’ – CAA AAC CTG AAT CAG TGC CTA CAC - 3’ | 0.5 M |  |
| SMPp26884S-MGB | 5’ - 6FAM – CTG CCA AAA GAG ATC AC – MGBNFQ - 3’ | 0.1 M |  |

^1^A GenBank BLAST of each primer/probe sequence resulted in 100% homology with every completely sequenced SARS strain currently available in the database (102 strains). Only the p26884S-MGB probe sequence showed any cross-homology to *Oryza sativa* chromosome 3 BAC OSJNBa0087M10 (accession # AC109602) and BAC OJ1523_A02 (accession # AC090874) and *Homo sapiens* chromosome 17, clone RP11-455O6 (accession # AC027601). Also, POL-R90 primer has a 26/27 base pair match with SARS GZ-C (accession # AY4979) and a 23/27 base pair match with SARS Sin3408L (accession # AY559097). However, the primers and probe for the SMP assay are a complete match to these two SARS strains.
