# Supplementary material for: Cynomolgus Macaque as an Animal Model for Severe Acute Respiratory Syndrome
Source: PLoS Med. 2006 Apr 18;3(5):e149. doi: 10.1371/journal.pmed.0030149 (PMC1435788; doi:10.1371/journal.pmed.0030149)
Supplement: Supplementary file 5 [file pmed.0030149.st002.doc]

Table S2: Testing of SARS real-time PCR assays.

Results of POL-MGB and SMP-MGB assays for samples spiked with SARS coronoavirus versus samples spiked with non-SARS coronaviruses.

| **Organism** | **POL-MGB** | **SMP-MGB** |
| --- | --- | --- |
| SARS: |  |  |
| Urbani  (CDC) | + | + |
| TOR 3 | + | + |
| TOR 7 | + | + |
|  |  |  |
| Coronaviruses: |  |  |
| MHV-A59 | - | - |
| MHV-JHM | - | - |
| MHV-2 | - | - |
| TGEV | - | - |
| HCV-OC43 | - | - |
